# Supplementary material for: Opposing Roles of FoxA1 and FoxA3 in Intrahepatic Cholangiocarcinoma Progression
Source: Int J Mol Sci. 2020 Mar 5;21(5):1796. doi: 10.3390/ijms21051796 (PMC7084256; doi:10.3390/ijms21051796)
Supplement: Supplementary file 1 [file ijms-21-01796-s001.pdf]

Figure S1

S1A

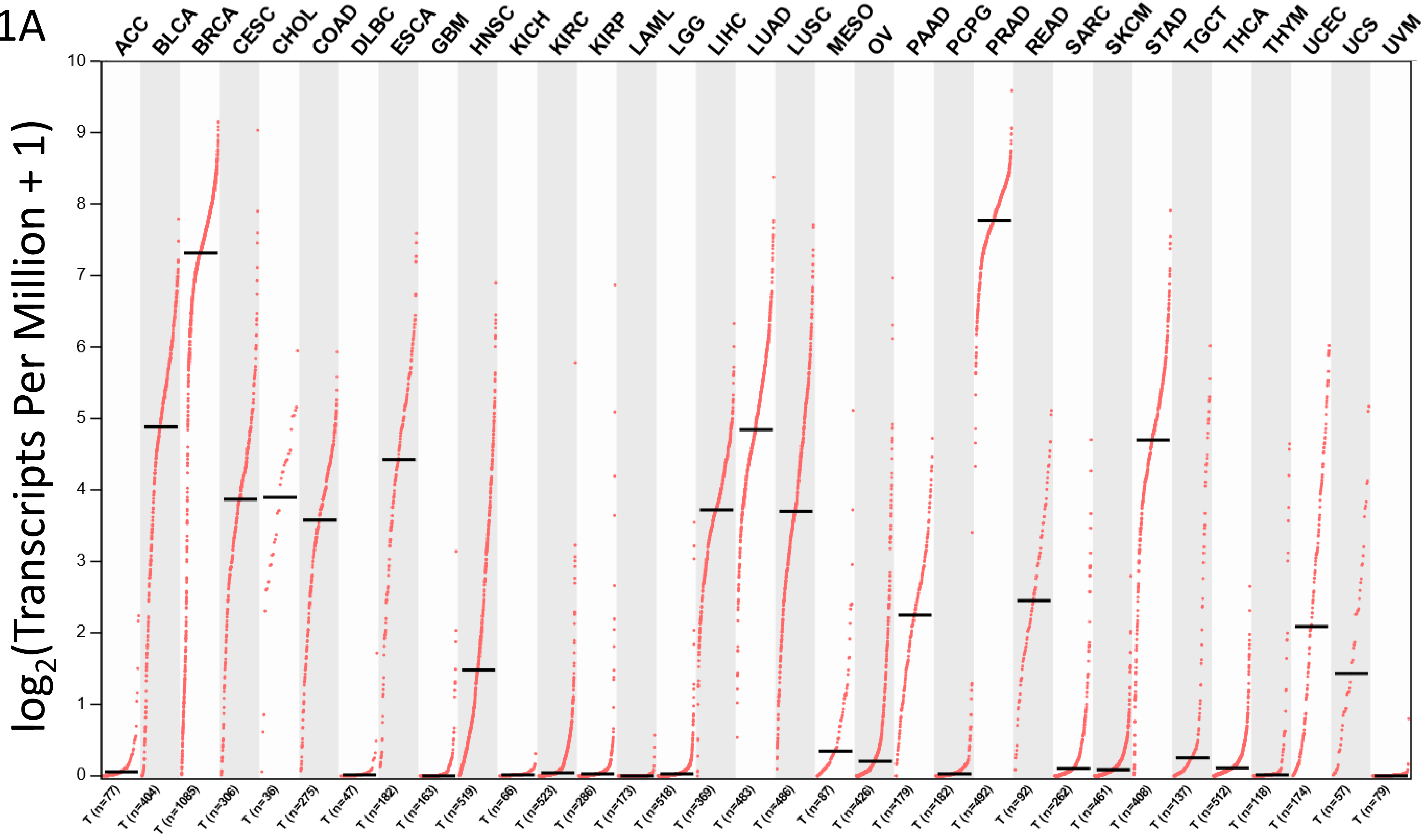

Figure S1

S1B

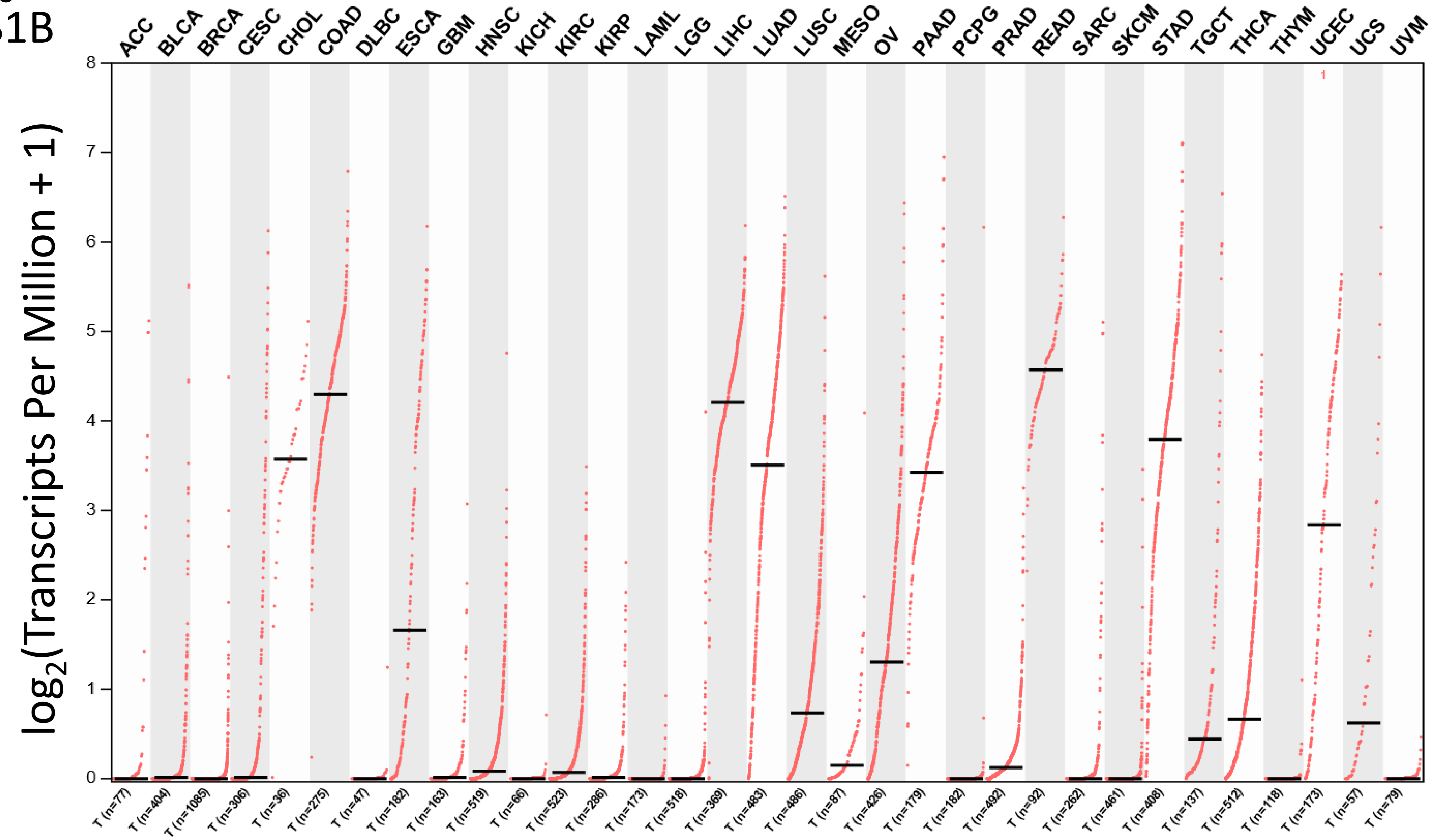

Figure S1

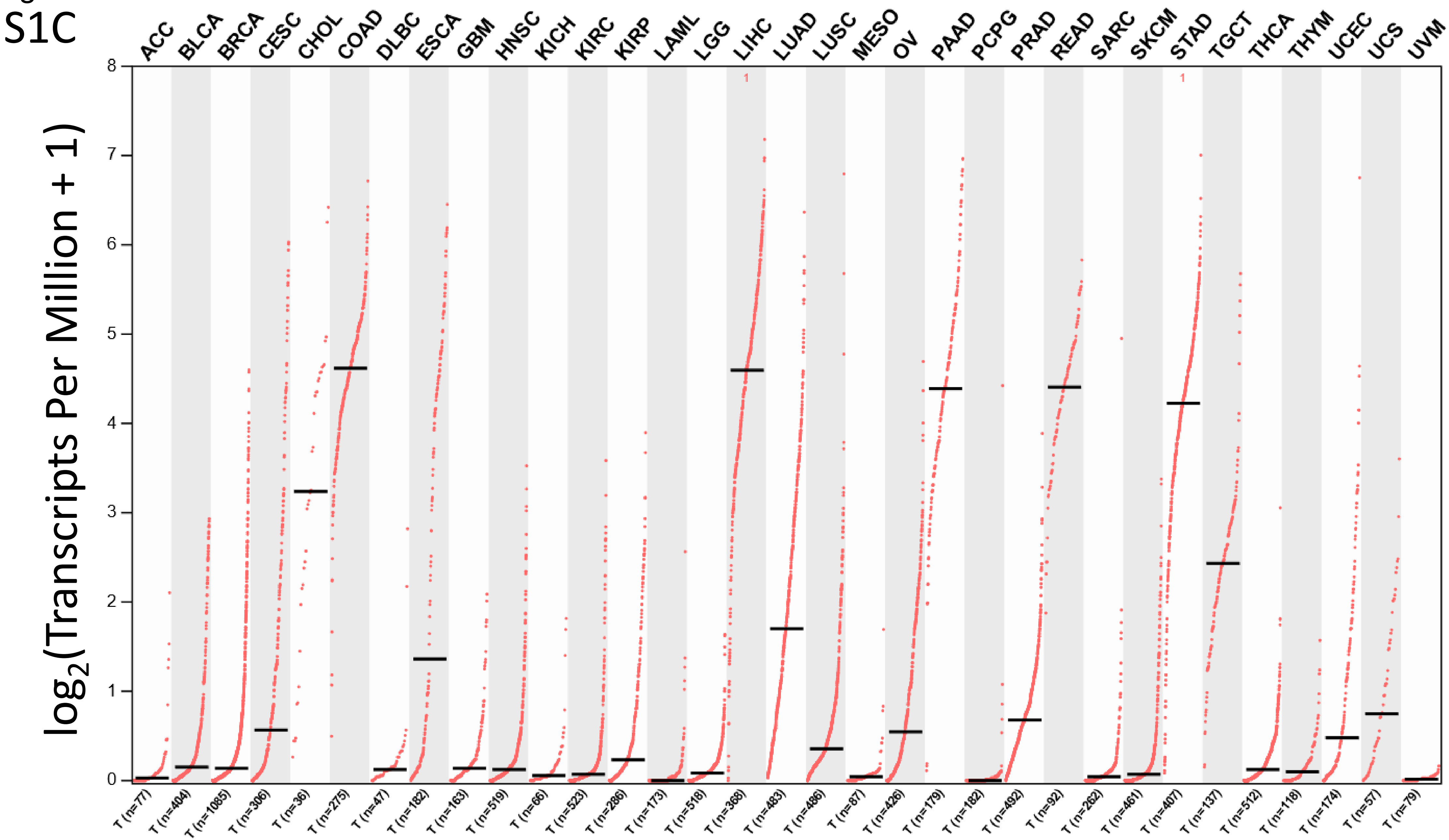
S1C
log2(Transcripts Per Million + 1)
ACC
BLCA
BRCA
CESC
CHOL
COAD
DLBC
ESCA
GBM
HNSC
KICH
KIRC
KIRP
LAML
LGG
LIHC
LUAD
LUSC
MESO
OV
PAAD
PCPG
PRAD
READ
SARC
SKCM
STAD
TGCT
THCA
THYM
UCEC
UCS
UVM
0
1
2
3
4
5
6
7
8
T (n=77)
T (n=404)
T (n=1085)
T (n=306)
T (n=36)
T (n=275)
T (n=47)
T (n=182)
T (n=163)
T (n=519)
T (n=66)
T (n=523)
T (n=286)
T (n=173)
T (n=518)
T (n=369)
T (n=483)
T (n=486)
T (n=87)
T (n=426)
T (n=179)
T (n=182)
T (n=492)
T (n=92)
T (n=262)
T (n=461)
T (n=407)
T (n=137)
T (n=512)
T (n=118)
T (n=174)
T (n=57)
T (n=79)

Table S1 Cancer types used in analysis of TCGA database

TCGA	Detail
ACC	Adrenocortical carcinoma
BLCA	Bladder Urothelial Carcinoma
BRCA	Breast invasive carcinoma
CESC	Cervical squamous cell carcinoma and endocervical adenocarcinoma
CHOL	Cholangio carcinoma
COAD	Colon adenocarcinoma
DLBC	Lymphoid Neoplasm Diffuse Large B-cell Lymphoma
ESCA	Esophageal carcinoma
GBM	Glioblastoma multiforme
HNSC	Head and Neck squamous cell carcinoma
KICH	Kidney Chromophobe
KIRC	Kidney renal clear cell carcinoma
KIRP	Kidney renal papillary cell carcinoma
LAML	Acute Myeloid Leukemia
LGG	Brain Lower Grade Glioma
LIHC	Liver hepatocellular carcinoma
LUAD	Lung adenocarcinoma
LUSC	Lung squamous cell carcinoma
MESO	Mesothelioma
OV	Ovarian serous cystadenocarcinoma
PAAD	Pancreatic adenocarcinoma
PCPG	Pheochromocytoma and Paraganglioma
PRAD	Prostate adenocarcinoma
READ	Rectum adenocarcinoma
SARC	Sarcoma
SKCM	Skin Cutaneous Melanoma
STAD	Stomach adenocarcinoma
TGCT	Testicular Germ Cell Tumors
THCA	Thyroid carcinoma
THYM	Thymoma
UCEC	Uterine Corpus Endometrial Carcinoma
UCS	Uterine Carcinosarcoma
UVM	Uveal Melanoma

Number of tumor sample
77
404
1085
306
36
275
47
182
163
519
66
523
286
173
518
369
483
486
87
426
179
182
492
92
262
461
408
137
512
118
174
57
79
